# The risk of psychiatric disorders among Finnish ART and spontaneously conceived children: Finnish population-based register study

**DOI:** 10.1007/s00787-019-01433-2

**Published:** 2019-11-04

**Authors:** E. Rissanen, M. Gissler, V. Lehti, A. Tiitinen

**Affiliations:** 1 Department of Obstetrics and Gynaecology, University of Helsinki, Helsinki, Finland; 2 Finnish Institute for Health and Welfare (THL), PO Box 30, 00271 Helsinki, Finland; 3 Research Centre for Child Psychiatry, University of Turku, Turku, Finland; 4 Division of Family Medicine, Department of Neurobiology, Care Sciences and Society, Karolinska Institute, Stockholm, Sweden; 5 Mental Health Unit, Finnish Institute for Health and Welfare (THL), Helsinki, Finland; 6 Department of Psychiatry, University of Helsinki and Helsinki University Hospital, Helsinki, Finland; 7 Department of Obstetrics and Gynaecology, University of Helsinki and Helsinki University Hospital, PO Box 140, 00029 Helsinki, Finland

**Keywords:** Assisted reproductive technique, Mental health, Psychiatric disorder, Register-based study

## Abstract

Evidence regarding the psychiatric morbidity of children born after Assisted Reproductive Techniques (ART) is inconsistent and limited. While normal mental well-being for ART children is usually reported, concerns are still being raised. Previous studies examine only some psychiatric disorders, but not all of them, ignore the impact of multiplicity, and limit the follow-up time to childhood. We examined all psychiatric diagnoses for singletons until their young adulthood. The aim was to study whether the risk of psychiatric disorders differs between ART and spontaneously conceived (SC) singletons until young adulthood. This retrospective Finnish population-based register study includes all ART and SC live-born children born in Finland during 1990–2013 and their hospital care in 1990–2014 (*n* = 1,425,975 of which 1,385,956, 97.2% were singletons). After excluding multiples, the final population included 17,610 ART and 1,368,346 SC singletons in 1990–2013 from the Finnish Medical Birth Registry. These data were linked to the Finnish Hospital Discharge Registry with the child’s and mother’s encrypted IDs. ART singletons had fewer psychiatric diagnoses (ART 10.2%, *n* = 1796, SC 12.0%, *n* = 164,408), but they received their diagnoses earlier (mean 8.3 years old, SD 5.0) than SC singletons (mean 10.5 years old, SD 5.7). After adjusting for confounding factors, ART singletons had an increased likelihood of getting a psychiatric diagnosis until young adulthood and the results were similar for boys (adjusted hazard ratios [aHR] = 1.16, 95% confidence interval (CI) 1.10–1.24) and girls (aHR = 1.25, 95% CI 1.16–1.35). We conclude that ART children receive their psychiatric diagnoses earlier than SC children, in particular during childhood and early adolescence. After adjusting for confounding factors ART children a slightly increased likelihood of any psychiatric diagnosis compared to SC controls.

**Electronic supplementary material:**

The online version of this article (10.1007/s00787-019-01433-2) contains supplementary material, which is available to authorized users.

## Introduction

The impact of assisted reproduction techniques (ART) on long-term mental health and psychiatric morbidity of offspring has received a little attention thus far, while the physical health of ART children has been studied more. Relatively few studies have examined mental well-being, especially until young adulthood. Since the use of ART is increasing, large-scale long-term data are required when studying especially rarer types of psychiatric disorders after assisted conception.

Previous studies have reported conflicting results of psychiatric outcomes among ART children. Some recent studies have concluded normal social, emotional, and cognitive functions, as well as mental well-being [1–6]. In an earlier Finnish study, increased risk for psychological and developmental disorders was presented [7]. Later, an increase in neither mental health nor cognitive developmental problems was evident for ART children aged 7–8 years [8]. Similarly, comparable mental development [9, 10] during childhood has been reported from other countries.

Neuropsychiatric disorders [e.g., Autism Spectrum Disorders (ASDs)] and intellectual disability are the most studied psychiatric problems in relation to ART treatment, but there is no consensus. Some studies have reported an increased risk of neuropsychiatric disorders [2, 11–13], while some have indicated no higher risk of ASD for ART children when compared to spontaneously conceived (SC) children [14–18]. No elevated risks of intellectual disability [18, 19] have been found for ART singletons.

Findings regarding behavioral and socio-emotional disorders [e.g., Attention Deficit and Hyperactivity Disorders (ADHD) and tic disorder] have also been inconsistent. Studies focusing on social problems and externalizing symptoms (e.g., aggression and other behavioral problems) have shown both comparable [2, 8], and higher prevalence [12] for ART children. Increased risks have particularly been observed related to hyperactive disorders [20] and tic disorder [21]. Indications for socio-emotional and behavioral problems among ART children have also been reported after 8 years of age [3].

The follow-up times of earlier studies have been limited mainly to childhood and data for adolescence and young adulthood are scarce. Thus, the psychiatric disorders with later onset have not been able to be studied. One study recently reported no differences between ART and SC children in the number of psychiatric diagnoses given at 3, 7, 14, and 18 years of age [22]. Other studies have reported conflicting results about psychiatric disorders manifesting until young adulthood among ART children [7, 23].

The mechanisms underlying ART as a possible causal factor for psychiatric disorders can only be speculated. Multiple pregnancies with higher risk of preterm birth and the associated higher morbidity have been a major obstacle from the early days of ART. Therefore, we excluded these children in our study. Laboratory procedures during fertilization and embryo culture could interfere with epigenetic processes during early embryo development. Furthermore, the health of the couple and underlying fertility background may increase pregnancy complications and thereby affect the child’s future health.

The aim of this population-based register study is primarily to investigate all psychiatric disorders of ART singleton children diagnosed in specialised health care until young adulthood. Our main objective is to assess the incidence and age at diagnosis of psychiatric disorders among ART and SC offspring and how this change by time; secondarily, we will assess whether the relationship of demographic and clinical factors with psychiatric disorders differs for ART and SC children.

## Methods

### Study population and design

This retrospective Finnish population-based register study comprises all ART (children conceived after In Vitro Fertilization [IVF], Intracytoplasmic Sperm Injection [ICSI], or Frozen Embryo Transfer [FET]) and SC live births born in Finland during 1990–2013 and their hospital care in 1990–2014, altogether 1,425,975 children. After excluding multiple births, our data include 17,610 ART singletons and 1,368,346 SC singletons. The number of children born after ovulation induction (OI) or intrauterine insemination (IUI) was available only from 2004 to 2013, and thus, these children (*n* = 10,455) were excluded from the analyses. The distribution of the study population is shown in Online Resource Fig. 1.

### Registries

The study data were established in the Finnish Institute for Health and Welfare (THL) and collected from the Finnish Medical Birth Registry (FMBR) and the Finnish Hospital Discharge Registry (FHDR, renamed the Care Registry for Health Care in 1994). In Finland, all pregnant women and their children are provided equal and comprehensive public health care including child health and maternity clinics and school health care and are referred to specialists if needed. The data in FMBR and FHDR are collected regularly alongside those visits in child health and maternity clinics, school health care, or specialized health care services.

The FMBR includes data on live births and stillbirths of at least 500 g birth weight or at least 22 weeks of gestational age, data on the infant (e.g., date of birth and sex) and mother (e.g., personal data of mother, previous pregnancies and deliveries, current pregnancy, and delivery) [24]. ART children were identified from the FMBR. From this register, we also obtained information on maternal age, parity, Socioeconomic Status (SES) based on maternal occupation, marital status and cohabitation, care during pregnancy, gestational age, and the number of fetuses.

The FHDR includes data on all discharges from inpatient care, in inpatient care in hospitals and health care centers, and all day surgeries (since 1994) and visits at specialized hospital outpatient care (since 1998) [25]. We included all diagnoses for mental, behavioral, and neurodevelopmental disorders (ICD-9: 290–319, ICD-10: F00–F99) [26], and examined the number of diagnoses and the age when the first diagnosis was received. The names of psychiatric diagnostic groups used in this study are shown in Online Resource Table 1. From the FHDR, we gathered children’s hospital care episodes in inpatient care in 1990–2014 and public hospital outpatient visits in 1998–2014 with diagnoses related to mental, behavioral, and neurodevelopmental disorders. We further collected information on the mothers’ hospital inpatient episodes with an ICD-8, ICD-9, or ICD-10 code for mental, behavioral and neurodevelopmental disorders as a primary diagnosis since 1969 until the childbirth. ICD-8 and ICD-9 codes were transformed to ICD-10 codes.

The links between mothers and children were available in the FMBR using the mother’s encrypted personal identification (ID) code including birth date and sex. This ID is given to each Finnish citizen after birth or permanent resident after immigration, and it is used widely in society (e.g., national registration system and healthcare services) [24], which makes the linkages complete.

### Variables

The variables and their classifications used were chosen based on existing literature and previous studies. Selected background factors were adjusted for in the analysis. The following variables were found to have impact on the outcome: child’s year of birth, mother’s age at delivery, parity, marital status, mother’s SES, mother’s psychiatric diagnoses in inpatient care, and prematurity.

We use the following age variables: child’s age recorded (mean weeks, SD) at the day of receiving the first diagnosis for mental, behavioral, and neurodevelopmental disorders and mother’s age at the date of delivery. The data on parity were categorized into three groups: no earlier deliveries (nulliparous), at least one earlier delivery (multiparous), and unknown. The data on mother’s marital status were classified into three groups: living with partner (married or cohabiting), living without partner (dissolved partnership, widowed, or single), and unknown. The data on maternal SES were based on occupation and categorized as four groups: upper white-collar worker, lower white-collar worker, blue-collar worker, and others (student, entrepreneur, unemployed, or housewife). The data on the mothers’ psychiatric diagnoses were classified into two groups: yes or no for having at least one psychiatric diagnosis in inpatient care. Prematurity (< 37 weeks) was based on the best clinical estimation of gestational age at birth, taking into account the last menstrual cycle (SC group) or day of embryo transfer (ART group).

### Statistical analyses

First, we tested differences in characteristic factors between ART and SC groups using the test of relative proportions and the *t* test. Second, all psychiatric diagnoses were examined and the number of diagnoses and the age when the first diagnosis was received were compared between ART and SC children with the relative frequency test and the *t* test. Statistical significance was defined as *p* < 0.001. The number of children with diagnoses of organic mental disorders (F00–09) was low, and these diagnoses are not reported separately. Unspecified mental disorders (F99) are included in the diagnostic category F90–98 due to their small numbers.

Next, the cumulative incidences of psychiatric diagnoses during the follow-up time 1990–2013 between ART and SC singletons were investigated: all F-diagnoses (F00–99) together, and each psychiatric diagnostic category separately for singleton boys and girls. Finally, the analyses were also performed by birth year. The study period was divided into three equal periods: 1990–1997, 1998–2006, and 2007–2013.

We used Cox regression to estimate Hazard Ratios (HRs) and differences in likelihood for psychiatric diagnoses of ART and SC children. Both unadjusted and adjusted HRs (aHR) with 95% Confidence Intervals (CIs) were calculated until first diagnoses or end of follow-up (31 December 2014). We had no data on deaths after 1 year neither on emigration. The aHRs were calculated for all singletons by including confounding factors: child’s sex, mother’s age at delivery, parity, mother’s marital status and SES, mother’s psychiatric inpatient care before childbirth (since 1969), and prematurity (< 37 weeks). Then, we examined the effect of confounding factors for singleton boys and girls separately.

## Results

The characteristics of mothers and their pregnancies are shown in Table 1. ART mothers were older (ART mean 33.8 years, SD 4.7; SC mean 29.7 years, SD 5.3; *p* < 0.0001) and had less often been diagnosed with a psychiatric disorder (ART 4.1%, SC 5.0%; *p* < 0.0001) than their SC controls. They also had higher SES (*p* < 0.0001) and were more often living with a partner (ART 94.3%, SC 89.9%; *p* < 0.0001). As expected, ART mothers were more often nulliparous (ART 66.3%, SC 40.2%; *p* < 0.0001).

**Table 1:** Characteristics of mothers and their pregnancies

		ART, *n* (%)^a^ *N* = 17 610	SC, *n* (%)^b^ *N* = 1 368 346	All, *n* (%)^c^ *N* = 1 385 956	*p* value
Mother’s age (mean years [SD])*		33.8 (4.7)	29.7 (5.3)	29.8 (5.3)	< 0.0001
Mother with psychiatric disorder		718 (4.1)	81 390 (5.0)	82 108 (5.9)	< 0.0001
Mother’s SES**	Upper white-collar worker	4606 (26.2)	209 041 (15.3)	213 647 (15.4)	< 0.0001
	Lower white-collar worker	7333 (41.6)	512 758 (37.5)	520 091 (37.5)	
	Blue-collar worker	2000 (11.4)	212 044 (15.5)	214 044(15.4)	
	Other***	3671 (20.8)	434 503 (31.8)	438 174 (31.6)	
Marital status****	Living with partner	16 601 (94.3)	1230 753 (89.9)	1 247 354 (90.0)	< 0.0001
	Living without partner	1006 (5.7)	134 584 (9.8)	135 590 (9.8)	
	Unknown	3 (0.0)	3009 (0.2)	3012 (0.2)	
Parity	Nulliparous	11 678 (66.3)	550 339 (40.2)	561 017 (40.6)	< 0.0001
	Multiparous	5916 (33.6)	814 605 (59.5)	820 521 (59.2)	
	Unknown	16 (0.1)	3402 (0.2)	3418 (0.2)	
Gestational age at birth (mean weeks [SD])		39.5 (2.0)	39.8 (1.7)	39.8 (1.7)	< 0.0001

*SC* spontaneously conceived, *ART* assisted reproductive techniques, *SD* standard deviation

*Age at the date delivery

**p < 0.001, × 2 test (4 × 2)

***Student, entrepreneur, unemployed, and housewife

****p < 0.001, × 2 test (7 × 2), living with partner: married or cohabiting, living without partner: dissolved partnership, widowed or single

^a^Percentages are calculated based on ART population, *n*/*N* (ART)

^b^Percentages are calculated based on SC population, *n*/*N* (SC)

^c^Percentages are calculated based on the total study population, *n*/*N* (All)

ART children had fewer psychiatric diagnoses (ART: 10.2%, *n* = 1796, SC: 12.0%, *n* = 164,408) (*p* < 0.0001), but they received the diagnoses at a younger age (mean 8.3 years; SD 5.0) than SC children (mean 10.5 years; SD 5.7) (*p* < 0.0001) (Table 2). The most common disorders for ART and SC girls were behavioral and emotional disorders with onset in childhood and adolescence (312 ART children, 3.6%; 25,952 SC children, 3.9%) and for ART and SC boys, disorders of psychological development (594 ART children, 6.6%; 46,055 SC children, 6.6%) (Table 3).

**Table 2:** Number of singleton children with at least one psychiatric diagnosis and the mean age of receiving the first diagnosis for mental, behavioural, and neurodevelopmental disorder

	ART	SC	All	*p* value
	*N* = 17,610 *N* (girls) = 8563 *N* (boys) = 9047	*N* = 1,368,346 *N* (girls) = 669 020 *N* (boys) = 699 326	*N* = 1,385,956 *N* (girls) = 677 583 *N* (boys) = 708 373	
All children				
* n* (%)^a^	1796 (10.2)	164 408 (12.0)	166 204 (12.0)	< 0.0001
Mean age (SD)^b^	8.3 (5.0)	10.5 (5.7)	10.4 (5.7)	< 0.0001
Girls				
* n* (%)^a^	740 (8.6)	70 594 (10.6)	71 334 (10.5)	< 0.0001
Mean age (SD)^b^	9.2 (5.6)	11.9 (5.9)	11.9 (5.9)	< 0.0001
Boys				
* n* (%)^a^	1056 (11.7)	93 814 (13.4)	94 870 (13.4)	< 0.0001
Mean age (SD)^b^	7.7 (4.5)	9.4 (5.2)	9.4 (5.2)	< 0.0001

*SC* spontaneously conceived, *ART* assisted reproductive techniques, *SD* standard deviation

^a^Percentages are calculated *n*/*N*

^b^The mean age of receiving the first diagnosis for mental, behavioral, and neurodevelopmental disorders

**Table 3:** The cumulative incidences of psychiatric diagnoses (F10–F99) for singletons during the follow-up period 1990–2013 in Finland

F-diagnosis	ART *N* (all singleton ART boys) = 9047 *N* (singleton ART boys with F-dg) = 1056 (11.7%)a	SC *N* (all singleton SC boys) = 699 326 *N* (singleton SC boys with F-dg) = 93 814 (13.4%)b	Crude HR (95% CI)	Adjusted HR^e^ (95% CI)
(a) Singleton boys				
All F-diagnosis*	11.66	13.34	1.09 (1.03–1.16)	1.16 (1.10–1.24)
F10–19	0.18	0.77	0.55 (0.33–0.89)	0.72 (0.43–1.20)
F20–29	0.21	0.34	1.47 (0.93–2.30)	1.48 (0.93–2.36)
F30–39	0.80	1.63	0.90 (0.72–1.14)	1.04 (0.82–1.32)
F40–49	1.34	2.10	1.06 (0.89–1.27)	1.52 (0.96–1.38)
F50–59	0.88	0.72	1.49 (1.19–1.86)	1.40 (1.12–1.74)
F60–69	0.09	0.20	0.85 (0.42–1.70)	0.99 (0.49–1.99)
F70–79	0.76	0.73	1.27 (1.00–1.61)	1.35 (1.06–1.72)
F80–89	6.57	6.59	1.15 (1.06–1.25)	1.17 (1.08–1.27)
F90–99	5.46	6.25	1.07 (0.98–1.17)	1.20 (1.10–1.31)
(b) Singleton girls				
All F-diagnosis*	8.63	10.51	1.16 (1.08–1.25)	1.25 (1.16–1.35)
F10–19	0.26	0.71	0.78 (0.51–1.59)	1.08 (0.71–1.65)
F20–29	0.20	0.33	1.29 (0.80–2.08)	1.40 (0.86–2.26)
F30–39	1.63	3.17	1.02 (0.87–1.21)	1.16 (0.98–1.37)
F40–49	2.18	3.43	1.15 (1.00–1.33)	1.28 (1.12–1.48)
F50–59	1.19	1.46	0.81 (0.67–9.99)	1.09 (0.89–1.32)
F60–69	0.19	0.37	1.27 (0.78–2.08)	1.40 (0.86–2.30)
F70–79	0.43	0.47	1.12 (0.81–1.56)	1.16 (0.84–1.61)
F80–89	2.74	2.83	1.13 (1.00–1.28)	1.20 (1.06–1.37)
F90–99	3.64	3.88	1.21 (1.08–1.35)	1.34 (1.20–1.50)

*SC* spontaneously conceived, *ART* assisted reproductive techniques, *HR* hazard ratio, *CI* confidence interval

*All F-diagnosis = F10–99

^a^Percentages are calculated based on population of singleton ART boys, *n*/*N* (singleton ART boys)

^b^Percentages are calculated based on population of singleton SC boys, *n*/*N* (singleton SC boys)

^c^Percentages are calculated based on population of singleton ART girls, *n*/*N* (singleton ART girls)

^d^Percentages are calculated based on population of singleton SC girls, *n*/*N* (singleton SC girls)

^e^Cox regressions, hazard ratios (HR) adjusted for confounding factors: year of birth, mother’s age at delivery, parity, marital status and SES, psychiatric inpatient care before childbirth (since 1969), and prematurity (< 37 weeks)

### Cumulative incidences of psychiatric diagnoses

**Fig. 1:**
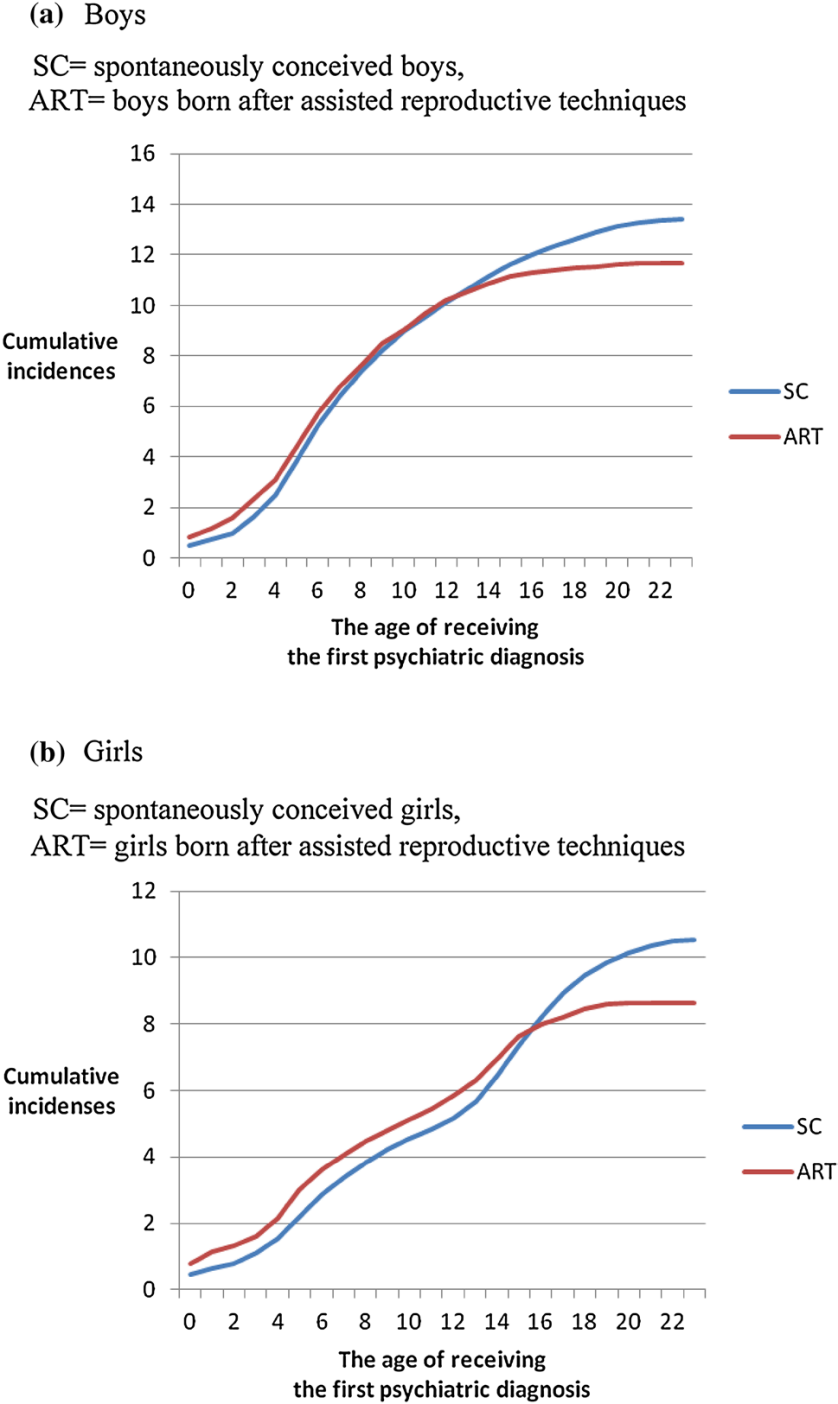
Cumulative incidences of psychiatric diagnoses (F10–F99) for singleton boys and girls born in 1990–2013. **a** Boys. *SC* spontaneously conceived boys, *ART* boys born after assisted reproductive techniques. **b** Girls. *SC* spontaneously conceived girls, *ART* girls born after assisted reproductive techniques

The cumulative incidences and unadjusted HRs of any psychiatric diagnosis (F10–F99) for singleton boys and girls born in 1990–2013 are shown in Fig. 1 and Table 3. In unadjusted analyses, the likelihood of being diagnosed for any psychiatric diagnosis was slightly increased for ART singletons; the HR was 1.09 (95% CI 1.03–1.16) for boys and 1.16 (95% CI 1.08–1.25) for girls.

After adjusting for background factors [child’s birth year and sex, mother’s age at delivery, parity, mother’s marital status and SES, and mother’s psychiatric inpatient care before childbirth and prematurity (< 37 weeks)], the likelihood of any psychiatric diagnosis during the follow-up time was slightly increased for both the singleton ART boys (aHR = 1.16, 95% CI 1.10–1.24) and girls (aHR = 1.25, 95% CI 1.16–1.35) (Table 3).

When examining background-adjusted hazard ratios by period, the increased likelihoods of any psychiatric diagnosis were found for ART children through the follow-up time when compared to SC children for all periods; for children born in 1990–1997 aHR = 1.18, 95% CI 1.10–1.27, in 1998–2006 aHR = 1.12, 95% CI 1.04–1.21, and in 2007–2013 aHR = 1.19, 95% CI 1.04–1.37 (Table 4). When regarding particular diagnostic categories, ART children born in 1990–1997 had increased likelihoods of disorders of psychological development (aHR = 1.21, 95% 1.08–1.35), and for behavioural and emotional disorders with onset in childhood and adolescence (aHR = 1.26, 95% CI 1.13–1.41). For ART children born in 1998–2006, increased likelihoods were found for schizophrenia and other non-affective psychoses (aHR = 3.80, 95% CI 1.74–8.33), for anxiety disorders (aHR = 1.39, 95% CI 1.11–1.75), and for disorders of psychological development (aHR = 1.14, 95% 1.03–1.27). For children born in 2007–2013, higher likelihoods were found for intellectual disability (aHR = 1.62, 95% CI 1.01–2.60), and for behavioural and emotional disorders with onset in childhood and adolescence (aHR = 1.38, 95% CI 1.11–1.72). The cumulative incidences by birth year are shown in Table 4.

**Table 4:** Cumulative incidences of psychiatric diagnoses (F10–F99) for singletons born in 1990–1997, 1998–2006, and 2007–2013

	ART	SC	Adjusted HR*** (95% CI)
(a) 1990–1997	*N* = 3331	*N* = 490,210	
All F-diagnoses*	18.6	19.2	1.18 (1.10–1.27)
F10–19	1.1	2.0	0.95 (0.69–1.33)
F20–29	0.8	0.9	1.28 (0.88–1.88)
F30–39	4.7	5.9	1.10 (0.95–1.28)
F40–49	5.0	6.0	1.14 (0.99–1.31)
F50–59	2.3	1.9	1.22 (0.99–1.50)
F60–69	0.5	0.7	1.23 (0.77–1.95)
F70–79	0.8	0.8	1.20 (0.86–1.67)
F80–89	7.0	6.1	1.21 (1.08–1.35)
F90–99	7.5	7.1	1.26 (1.13–1.41)
(b) 1998–2006	*N* = 7906	*N* = 485,958	
All F-diagnoses*	12.3	12.2	1.12 (1.04–1.21)
F10–19	0.0	0.1	**
F20–29	0.1	0.1	3.80 (1.74–8.33)
F30–39	0.7	0.8	1.12 (0.72–1.75)
F40–49	1.7	1.6	1.39 (1.11–1.75)
F50–59	1.0	0.9	1.06 (0.81–1.40)
F60–69	0.1	0.1	1.68 (0.68–4.13)
F70–79	0.8	0.8	1.12 (0.84–1.49)
F80–89	6.3	6.1	1.14 (1.03–1.27)
F90–99	6.0	6.4	1.06 (0.95–1.18)
(c) 2007–2013	*N* = 6373	*N* = 392,178	
All F-diagnosis*	3.2	2.9	1.19 (1.04–1.37)
F10–19	0.0	0.0	**
F20–29	0.0	0.0	**
F30–39	0.0	0.0	6.51 (0.78–54.02)
F40–49	0.2	0.1	1.68 (0.92–3.07)
F50–59	0.4	0.3	1.01 (0.67–1.52)
F60–69	0.0	0.0	**
F70–79	0.3	0.2	1.62 (1.01–2.60)
F80–89	1.5	1.6	1.04 (0.85–1.27)
F90–99	1.3	1.0	1.38 (1.11–1.72)

*SC* spontaneously conceived, *ART* assisted reproductive techniques, *HR* hazard ratio, *CI* confidence interval

*All F-diagnosis = F10–99

**Not applicable due to low number of cases

***Cox regression, hazard ratios (HR) adjusted for confounding factors: year of birth, child’s sex, mother’s age at delivery, parity, mother’s marital status and SES, mother’s psychiatric inpatient care before childbirth (since 1969), and prematurity (< 37 weeks)

All confounding factors reached statistical significance when comparing ART and SC singletons, excluding parity for boys (data not shown). Mother’s psychiatric inpatient care before childbirth, prematurity, and low SES were the strongest predictors for any psychiatric diagnosis for singletons. Mother’s psychiatric inpatient care before childbirth correlated stronger for girls than boys, and low SES correlated stronger for boys than girls (data not shown).

## Discussion

This register-based study compared psychiatric morbidity between ART and SC singleton children during childhood, adolescence, and young adulthood. Our main finding was that ART children have fewer psychiatric diagnoses than SC children, but they receive their diagnoses younger. However, after adjusting for background factors, ART singletons had a slightly higher likelihood of any psychiatric diagnosis than SC children. This excess risk also remained similar over time. The largest differences when comparing ART and SC singletons born in 1990–1997 were found for disorders of psychological development and for behavioural and emotional disorders with onset in childhood and adolescence. Correspondingly, singletons born in 1998–2006 had largest differences in schizophrenia and other non-affective psychoses, anxiety disorders, and disorders of psychological development, and for singletons born in 2007–2013 for behavioural and emotional disorders with onset in childhood and adolescence, and intellectual disability. Our results partly challenge the current view, since the most previous studies report on equal mental well-being for ART and SC children in general [4, 6, 21, 27].

We found that ART children received their diagnoses on average 2 years younger than SC children. This can be caused by differences in use of health services. The SES impacts on health, cognitive, and socio-emotional outcomes in children, and a variety of mechanisms related to children and their parents are found to link SES to child well-being [28]. According to Larsson et al. [29] and Lehti et al. [30], maternal SES does not affect the risk of childhood autism, whereas Rai et al. [31] found an association between low SES and elevated risk of ASD. In our study, ART mothers, compared to SC controls, had higher SES and they were more often married, but their offspring still carried a higher risk of receiving any psychiatric diagnosis and a diagnosis for disorders of psychological development (including, e.g., ASD) in early life. This may be due to people with higher SES typically living in cities and large towns where health care services are easily available. ART children might be readily referred to health care, and thus are more likely to receive a psychiatric diagnosis and treatment for their psychiatric problems. Our data were based on the use of hospital services, and parents of ART children might seek health care more readily due to their child’s special position in their families as a precious child. In our study, ART mothers were also more often nulliparous, and it could be that the first and precious child, as ART children typically are, faces higher expectations from their parents. More protective parenting in ART families has been reported previously [12], so ART children might use heath care services more often.

ART children had an increased risk of psychiatric disorders [including, for example Attention Deficit Disorder (ADD), ADHD, and tic disorder] with onset in childhood and adolescence in adjusted analyses. Earlier studies have reported that ART children experience more ADD [32], ADHD, hyperactive disorders [20], tic disorders [21], and behavioral problems in general [12], but there are also findings of comparable outcomes between ART and SC groups [2, 8]. Most of these studies have examined all children, but not singletons separately. The youngest ART children had higher risk for intellectual disability, and behavioural and emotional disorders with onset in childhood and adolescence, which are typically diagnosed during early life. The oldest ART children had higher risk also for behavioural and emotional disorders with onset in childhood and adolescence, and additionally of disorders of psychological development, but not for other categories.

The duration of follow-up varied between participants. Those with longest follow-up time were adults by the end of the study and had certainly passed the typical age of onset for childhood disorders whereas those with shortest follow-up were still children and could not have been diagnosed with disorders which have a later onset. By dividing the participants in three groups by their birth year, it was easier to compare the association between ART and different diagnostic categories.

Psychological development and psychiatric morbidity in children and adolescents are reportedly influenced by the child’s sex [2, 33]. Early onset disorders (e.g., ASD and behavioral problems) show a marked male preponderance, while adolescence-onset disorders, such as anxiety and depression, show a marked female preponderance [33]. ART boys showed lower, but ART girls higher, levels of cognitive problems than their sex-matched SC controls, whereas no sex differences in mental health or developmental outcomes were found between ART girls and ART boys in a questionnaire-based study [8]. In our study, we found small sex differences when we examined psychiatric diagnostic categories separately. Compared to singleton SC controls, singleton ART boys had a higher risk of behavioral syndromes associated with physiological disturbances and physical factors, and intellectual disability, while singleton ART girls had a higher risk of anxiety disorders. However, these results are considered as clinically insignificant.

Parental age associates with the child’s psychiatric morbidity [34]. Both advanced maternal [35] and paternal age [34] are linked to higher risks of ASD and other pervasive developmental disorders (PDDs), but not with bipolar disorders [36, 37]. Younger parental age also generally associates with increased rates of a child’s psychopathology, especially PDDs [35], behavioral syndromes, and psychosis in youth [34]. In contrast, Larsson et al. [29] found no association between parental age and a child’s risk of ASD. With our data, ART mothers were a few years older than SC mothers at delivery, and the general likelihood of psychiatric disorders for their offspring were higher.

Parental psychiatric morbidity is a risk factor of offspring psychiatric disorders and the risks—for, for example, anxiety, depressive, and conduct disorders—are even greater if both parents have disorders [38]. Offspring of depressed parents are found to be at a higher risk of anxiety disorders, major depression, and substance dependence [39]. Associations have also been reported for parental alcohol-use disorders, depression, anxiety disorders, or social phobia and offspring social phobia [40], as well as parental schizophrenia-like psychosis or affective disorders and offspring ASD [29, 41]. In our study, ART mothers had less hospital-treated psychiatric disorders than SC mothers; this could partly explain our finding that ART children had fewer psychiatric diagnoses before adjustments.

It is challenging to identify any specific underlying mechanism or explanation for our main result that ART children received psychiatric diagnoses earlier than SC children and have slightly increased likelihood of such diagnoses compared to SC controls. Psychiatric disorders have different etiologies affected by biological, psychological, social, and epigenetic factors, and those factors are poorly known so far. When discussing ART offspring, they are obviously desired children, and their parents supposedly are more alert to any kind of symptoms of their child. ART parents have typically higher SES, which potentially plays a role in parent’s health knowledge as well as their sensitivity and financial possibility to use health care services. Therefore, ART children may be followed at younger ages and may use health care services more often. This explanation is supported by the earlier finding that ART parents show more protection of their children throughout their development [12]. Previous reports also suggest that stressful and mentally challenging-assisted conception procedures affect adaption to the parental role and the way that parents view their child, which naturally affects the child–parent relationship and child development [12, 42, 43]. However, we cannot rule out that ART methods as such could increase the risk for psychiatric morbidity.

Our study has weaknesses and limitations. The increased likelihood of psychiatric diagnoses in ART children remained over time, but the follow-up time may not be long enough for the youngest cohorts. The diagnoses of schizophrenia and other non-affective psychoses, substance use disorders, and personality disorders are typically received at older ages. We lacked information on background factors, such as paternal age, mother’s somatic diseases, and substance use during pregnancy, nor did we have data on family circumstances (e.g., parental divorces), child–parent relationship, region, or municipality for all cohort members in our register-based data. The impact of infertility itself, the specific method of ART treatment, and use of ovum or sperm donation could not be analyzed, since these data were not collected in the FMBR during the follow-up time of this study. OI/IUI treatments done in 1990–2003 were not possible to exclude, but the number of those OI/IUI treatments was so small that it did not effect on the results. Therefore, it may not be justified to draw any firm conclusions on the impact of genetic or embryonic programming on the development of ART children. Data on primary health care was unavailable, but children in those services typically experience mild psychiatric disorders and are not in need of specialized health care services. The same applies for mothers, for which we only had information on hospital care. Finally, we had no information on children’s deaths after the age of 1 year or on international migration, which was a limitation regarding our statistical methods. Finally, differences in early censoring causes bias in Cox regressions [44]. Since ART children received their diagnoses earlier than SC, our hazard ratios are overestimating child’s risk for psychiatric diagnoses.

Despite these limitations, our study overcomes most methodological problems of former studies and fulfils gaps in the literature with large data consisting of all psychiatric diagnoses until the young adulthood of ART and SC children and examines singletons separately. A strength of our study is that it is based on registers and the data used are large and high-class. In Finland, all pregnant women and their children are provided equal and comprehensive public health care including high-quality child health and maternity clinics and school health care, and are referred to specialists if needed. In addition, public health care is mainly free for children until the age of 18 years. The diagnoses used in this study were set by specialists of child, adolescent, or adult psychiatry, or child neurology, and based on thorough and multi-professional investigations. We controlled for preterm birth as a significant confounder, and crude rates show that preterm birth is a risk factor of receiving a psychiatric diagnosis for both boys and girls, even for singletons.

## Conclusion

In our study, ART children received the diagnoses earlier than SC children and had slightly increased likelihood of any psychiatric diagnosis compared to SC controls and the difference remained through the follow-up time. Our results challenge earlier findings on similar psychosocial and mental well-being, normal cognitive outcomes, and equal frequencies of psychiatric disorders among ART children [3, 4]. Further studies with longer follow-up data are needed to study specific psychiatric diagnostic categories among ART children in particular and to be able to draw wider conclusions.

## Electronic supplementary material

Below is the link to the electronic supplementary material.

Supplementary file1 (PDF 160 kb)

Supplementary file2 (PDF 75 kb)
