# Supplementary material for: The risk of psychiatric disorders among Finnish ART and spontaneously conceived children: Finnish population-based register study
Source: Eur Child Adolesc Psychiatry. 2019 Nov 4;29(8):1155–64. doi: 10.1007/s00787-019-01433-2 (PMC7369258; doi:10.1007/s00787-019-01433-2)
Supplement: Supplementary file 1 — Supplementary file1 (PDF 160 kb) [file 787_2019_1433_MOESM1_ESM.pdf]

Article Title: The risk of psychiatric disorders among Finnish ART and spontaneously conceived children: Finnish population-based register study.

Journal: European Child & Adolescent Psychiatry

Corresponding author: MD Essi Rissanen, Doctoral student, Department of Obstetrics and Gynaecology, University of Helsinki, Helsinki, Finland. Tel: +358443035727 E-mail: essi.rissanen@helsinki.fi

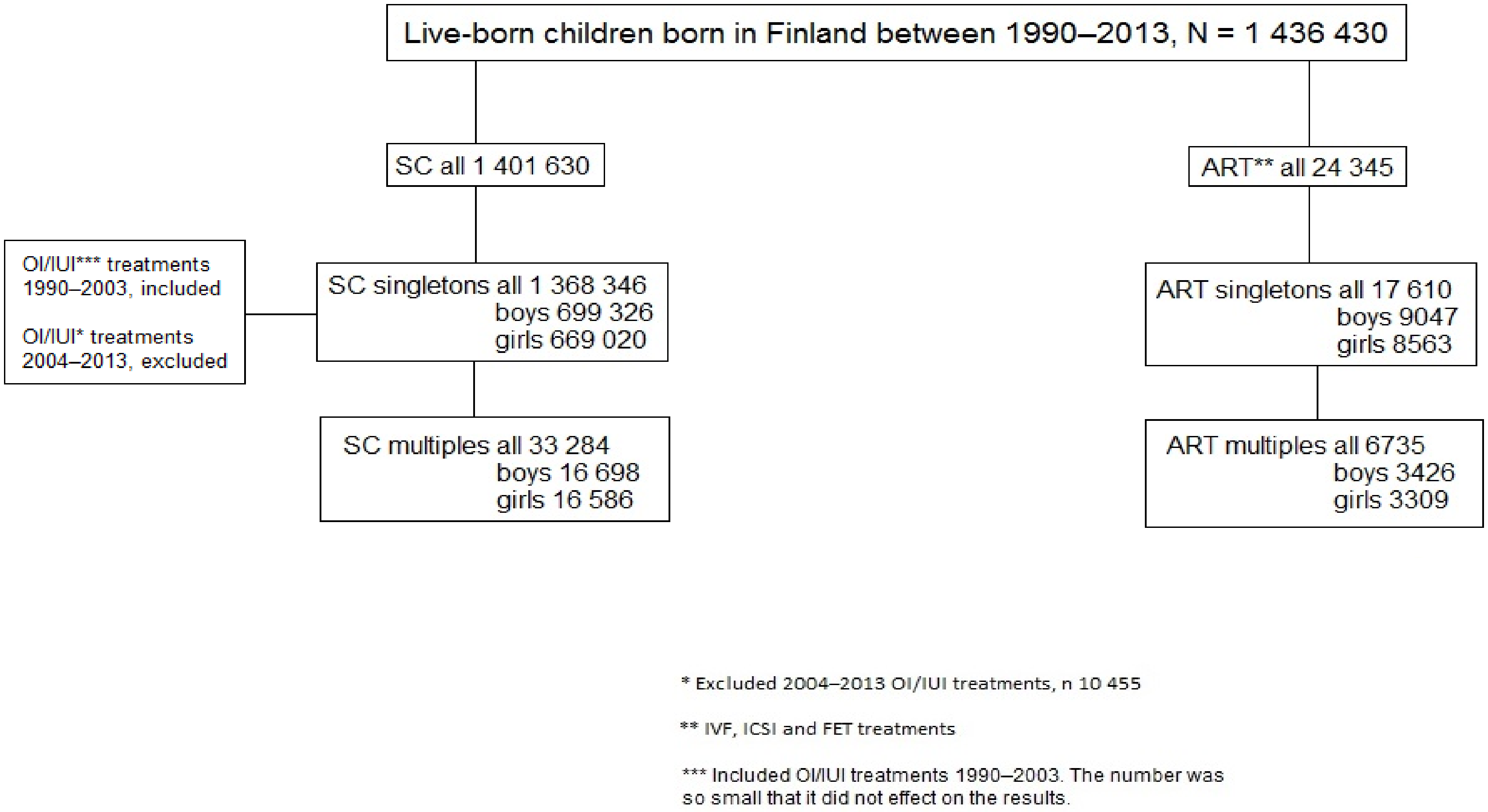

Online Resource Fig. 1 Distribution of the study population. SC= Spontaneously Conceived, ART= Assisted Reproductive Techniques, OI= Ovulation Induction, IUI= Intrauterine Insemination, IVF= In Vitro Fertilisation, ICSI= Intracytoplasmic Sperm Injection, FET= Frozen Embryo Transfer
