# Supplementary material for: The risk of psychiatric disorders among Finnish ART and spontaneously conceived children: Finnish population-based register study
Source: Eur Child Adolesc Psychiatry. 2019 Nov 4;29(8):1155–64. doi: 10.1007/s00787-019-01433-2 (PMC7369258; doi:10.1007/s00787-019-01433-2)
Supplement: Supplementary file 2 — Supplementary file2 (PDF 75 kb) [file 787_2019_1433_MOESM2_ESM.pdf]

Article Title: The risk of psychiatric disorders among Finnish ART and spontaneously conceived children: Finnish population-based register study.

Journal: European Child & Adolescent Psychiatry

Corresponding author: MD Essi Rissanen, Doctoral student, Department of Obstetrics and Gynaecology, University of Helsinki, Helsinki, Finland. Tel: +358443035727 E-mail: essi.rissanen@helsinki.fi

Online Resource Table 1 Diagnostic groups

Diagnostic group	ICD-10 Code (Version 2017, Chapter V, Mental, behavioural and neurodevelopmental disorders)	ICD-10 Classification (Version 2017, Chapter V, Version 2017, Chapter V, Mental, behavioural and neurodevelopmental disorders)	Subgroups
Organic mental disorders	F01–F09	Mental disorders due to known physiological conditions	F01 Vascular dementia, F02 Dementia in other diseases classified elsewhere, F03 Unspecified dementia, F04 Amnestic disorder due to known physiological condition, F05 Delirium due to known physiological condition, F06 Other mental disorders due to known physiological condition, F07 Personality and behavioural disorders due to known physiological condition, F09 Unspecified mental disorder due to known physiological condition
Substance use disorders	F10–F19	Mental and behavioural disorders due to psychoactive substance use	F10 Alcohol-related disorders, F11 Opioid-related disorders, F12 Cannabis-related disorders, F13 Sedative-, hypnotic-, or anxiolytic-related disorders, F14 Cocaine-related disorders, F15 Other stimulant-related disorders, F16 Hallucinogen-related disorders, F17 Nicotine dependence, F18 Inhalant-related disorders, F19 Other psychoactive substance-related disorders
Schizophrenia and other non-affective psychoses	F20–F29	Schizophrenia, schizotypal, delusional, and other non-mood psychotic disorders	F20 Schizophrenia, F21 Schizotypal disorder, F22 Delusional disorders, F23 Brief psychotic disorders, F24 Shared psychotic disorder, F25 Schizoaffective disorders, F28 Other psychotic disorder not due to a substance or known physiological condition, F29 Unspecified psychosis not due to a substance or known physiological condition
Affective disorders	F30–F39	Mood (affective) disorders	F30 Manic episode, F31 Bipolar disorder, F32 Major depressive disorder, single episode, F33 Major depressive disorder, recurrent, F34 Persistent mood [affective] disorders, F39 Unspecified mood [affective] disorder
Anxiety disorders	F40–F49	Anxiety, dissociative, stress-related, somatoform and other nonpsychotic mental disorders	F40 Phobic anxiety disorders, F41 Other anxiety disorders, F42 Obsessive-compulsive disorder, F43 Reaction to severe stress, and adjustment disorders, F44 Dissociative and conversion disorders, F45 Somatoform disorders, F48 Other nonpsychotic mental disorders
Behavioural syndromes associated with physiological disturbances and physical factors	F50–F59	Behavioural syndromes associated with physiological disturbances and physical factors	F50 Eating disorders, F51 Sleep disorders not due to a substance or known physiological condition, F52 Sexual dysfunction not due to a substance or known physiological condition, F53 Puerperal psychosis, F54 Psychological and behavioural factors associated with disorders or diseases classified elsewhere, F55 Abuse of non-psychoactive substances, F59 Unspecified behavioural syndromes associated with physiological disturbances and physical factors
Personality disorders	F60–F69	Disorders of adult personality and behaviour	F60 Specific personality disorders, F63 Impulse disorders, F64 Gender identity disorders, F65 Paraphilias, F66 Other sexual disorders, F68 Other disorders of adult personality and behaviour, F69 Unspecified disorder of adult personality and behaviour
Intellectual disability	F70–F79	Intellectual disabilities	F70 Mild intellectual disabilities, F71 Moderate intellectual disabilities, F72 Severe intellectual disabilities, F73 Profound intellectual disabilities, F78 Other intellectual disabilities, F79 Unspecified intellectual disabilities
Disorders of psychological development	F80–F89	Pervasive and specific developmental disorders	F80 Specific developmental disorders of speech and language, F81 Specific developmental disorders of scholastic skills, F82 Specific developmental disorder of motor function, F84 Pervasive developmental disorders, F88 Other disorders of psychological development, F89 Unspecified disorder of psychological development
Behavioural and emotional disorders with onset in childhood and adolescence	F90–F98	Behavioural and emotional disorders with onset usually occurring in childhood and adolescence	F90 Attention-deficit hyperactivity disorders, F91Conduct disorders, F93 Emotional disorders with onset specific to childhood, F94 Disorders of social functioning with onset specific to childhood and adolescence, F95 Tic disorders, F98 Other behavioural and emotional disorders with onset usually occurring in childhood and adolescence
Unspecified mental disorder	F99	Unspecified mental disorder	F99 Mental disorder, not otherwise specified
